# Aberrant Driving Behaviour, Risk Involvement, and Their Related Factors Among Taxi Drivers

**DOI:** 10.3390/ijerph15081626

**Published:** 2018-08-01

**Authors:** Javadreza Vahedi, Afshin Shariat Mohaymany, Zahra Tabibi, Milad Mehdizadeh

**Affiliations:** 1 Transportation Planning, School of Civil Engineering, Iran University of Science & Technology, Tehran 16846-13114, Iran; vahedi@ub.ac.ir (J.V.); milad_mehdizadeh@ymail.com (M.M.); 2 Psychology, Department of Clinical Psychology, Ferdowsi University of Mashhad, Mashhad 91779-48974, Iran; tabibi@um.ac.ir

**Keywords:** aberrant driving behaviour, accident involvement, taxi drivers, driver behaviour questionnaire

## Abstract

The current study aims to investigate the aberrant driving behaviour and risk involvement of Iranian taxi drivers. The sample comprised 405 Iranian taxi drivers, who were recruited with a cross-sectional design, using a self-completion questionnaire survey during October and November 2016. We contribute to the literature by understanding how and to what extent the socioeconomic, demographic, driving, and aberrant driving behaviours influence risk involvement (accident involvement and traffic tickets). The validated 27-item Driver Behaviour Questionnaire (DBQ) was applied to measure aberrant driving behaviour. The results from valid observations (*n* = 381) explored a four-factor solution (including errors, ordinary violations, lapses, and aggressive violations) of the DBQ. The results also showed that being a single driver, having a high annual driving mileage, and a high number of daily taxi trips were positively associated with accident involvement. Furthermore, there was a positive correlation between the more ordinary violations and aggressive violations and accident involvement. Establishing better training and qualification mechanisms for taxi drivers could be considered by traffic safety experts in order to reduce ordinary and aggressive violations.

## 1. Introduction

A growing interest has emerged in exploring the principal human factors of the driving behaviour in road transport studies. Road traffic accidents are recognised as a major source of fatality in the world. Several studies have shown that human factors play a critical role in road traffic accidents [1,2,3].

However, little is known about the aberrant driving behaviour of the taxi drivers, despite its importance [4,5,6,7], particularly among taxi drivers in the Middle Eastern context. Firstly, taxi drivers drive in a work-related context [8]. A small body of studies have investigated an occupational context [6,9], and most of them have examined the driving behaviour among the general driving population. Taxi drivers are mostly males and spend most of their time in urban traffic. In addition, the taxi is recognised as a private mode of transportation in western countries, while taxi services are seen as a mode of public transport (PT) in some eastern countries like Iran. This type of taxi has become the main dimension of the integrated transport system in Iranian cities and is playing an important role in passengers’ daily urban travel. Taxi drivers do not have to undergo specific training and qualification in Iran. Any person with an ordinary driver’s license and no major criminal record can be employed as a taxi driver. As taxi fares as a public transport mode are relatively low in Iran, taxi drivers often comprise the low-income strata of society. Because of the higher workload and spending greater time in traffic congestions, these drivers usually commit more risky driving compared with the general driving population. Furthermore, the taxi industry in Iran, as an organisation related to municipalities, is responsible for the management of taxis by taking measures such as determining the number of required taxis in each traffic analysis zone (TAZ) based on demand.

Most previous studies have been conducted in countries with good traffic safety performance. There might be an increase in the negative consequences of road traffic accidents in regions with low transport safety performance in the Middle East context, such as Iran [10,11]. For example, the global status report on road safety shows that the share of pedestrians in traffic-related mortalities in Iran is 23% [12]. Surprisingly, the national statistics of Iran show a high rate of fatality and injuries in road traffic accidents between taxis and young pedestrians [13]. Dalziel and Job [14] also found that taxi–pedestrian collisions were ranked as the fifth cause of all traffic accidents in Australia. A study by Shi et al. [4] revealed that passengers and police appear to have a very negative impression of the taxi drivers’ driving behaviours in Beijing, China. Hence, understanding how demographic variables (e.g., age), socioeconomic characteristics (e.g., educational background and car ownership status), and the driving experiences of taxi drivers are related to their aberrant driving behaviour and risk involvement, could be interesting in countries with a similar traffic safety performance and work-related context, such as Iran. 

Given the importance of public health in occupational contexts, the current study aims to overcome this deficiency by investigating the aberrant driving behaviour and risk involvement of Iranian taxi drivers. For this purpose, we used the information of 405 Iranian taxi drivers, recruited with a cross-sectional design, using a self-completion questionnaire survey during October and November 2016. We contribute to the literature by (1) identifying the principal dimensions of aberrant driving behaviour (Driver Behaviour Questionnaire [DBQ] factors) and confirming the factor structure of the DBQ among taxi drivers; (2) exploring socioeconomic, demographic, and driving factors, explaining the identified DBQ factors; and (3) understanding how and to what extent the socioeconomic, demographic, driving, and identified DBQ factors influence risk involvement (accident involvement and receiving traffic tickets). 

The remaining sections are organised as follows: Firstly, a review of the literature is presented in the following section. Secondly, we describe the participants, data, and statistical procedure used in this study. Thirdly, the results of the statistical analysis used in this study are discussed. Fourthly, an in-depth discussion of the results is provided. The paper is concluded by summarising the key findings and offering planning suggestions.

## 2. Literature Review

A critical review of previous studies showed that (1) most of the efforts were focussed on identifying the principal dimensions of the DBQ, (2) a few studies have examined the determinants of the identified DBQ factors through demographic variables (e.g., age and gender) and driving characteristics (e.g., annual mileage), and (3) several papers have investigated the role of the DBQ factors, as well as the demographic variables (e.g., age, gender) and driving characteristics in risks, including in accident involvement and receiving traffic tickets. We classify the review based on the aforesaid three findings. Then, the study’s contribution and research gaps are highlighted at the end of this section.

### 2.1. The Driver Behaviour Questionnaire

The Driver Behaviour Questionnaire is one of the instruments widely used to measure the human factors related to driving (i.e., aberrant driving behaviour) in different parts of the world [15]. The DBQ has played a prominent role in the realm of traffic psychology. The notion traces back to 1990, when Reason and colleagues [16] represented the DBQ’s first version, by including 50 items in an instrument. The authors explored three principal factors (violations, dangerous errors, and relatively harmless lapses) for this instrument. Later, the DBQ was abridged or extended to cover new driving factors (e.g., aggressive violation) [17,18,19,20,21,22]. Several studies have applied the 28- or 27-item version of the DBQ in recent years. The DBQ’s extended version has been mostly reported by a four-factor solution, including ordinary or rule violations, aggressive violations, errors, and lapses [23,24,25,26]. Violations refer to a driver’s intention to infringe on the regulations of safe driving (e.g., overtaking a slow driver on the inside). Aggressive violations are hostile motivations for aggressive driving (e.g., getting angry at a driver and expressing your anger in some ways). Errors refer to misjudgements and observational failures in driving (e.g., failing to notice that pedestrians are crossing when turning into a side street); while lapses refer to limitations in memory and attention (e.g., hitting something overlooked when reversing). 

### 2.2. Correlates of the Driver Behaviour Questionnaire Factors 

Gender and age are the demographic factors that are reported to be correlated with the DBQ factors. Mesken et al. [27] found that men report fewer lapses and more violations than women do. Shi et al. [28] also showed that aggressive violation behaviours in male drivers are more common compared with female drivers. On the other hand, the authors found that male drivers make fewer errors and violations. Furthermore, Rimmö and Hakamies-Blomqvist [29] reported that males and females had similar results in the DBQ structure. Batool and Carsten [30] also found no significant gender differences in the violation score. Sullman et al. [23] indicated that younger drivers tend to have higher ordinary and aggressive violation. Batool and Carsten [30] found that younger drivers display more dangerous driving behaviours. Among other variables, some studies found that a high annual mileage, greater violations, and fewer lapses were correlated [16,23]. Batool and Carsten [30] found that middle-income group drivers were more likely to display aggressive driving behaviour. Furthermore, de Winter and Dodou [15], through a meta-analysis, showed that age and annual mileage could be important correlates of errors and violations. The authors concluded that younger drivers tend to have more violations and error scores, while more driving exposure was positively associated with violations and errors. 

### 2.3. Predictors of the Risks (Accident Involvement) 

Several studies have shown that older drivers have a lower rate of accident involvement [23,31], while an increased annual mileage (or driving exposure) escalate accident risks [31,32]. However, studies have not found any definitive and significant association between the driver’s gender, age, annual mileage, level of education, and accident risks, including accident involvement and receiving traffic tickets [24,33]. In addition to accident involvement, traffic tickets can be used as a risk indicator. A traffic ticket is the primary means of traffic law enforcement. Both types of tickets (either by a police officer or by traffic cameras) have been included in this study.

Regarding the relationship between the DBQ factors and accident risks, studies have found that ordinary violations [23,24,34,35,36,37], aggression [35], and pushing-speeding [38], as well as lapses and errors [39] are related to accident risks. For instance, Gras et al. [24] found that higher violation scores were positively related to accident involvement. Rowe et al. [36] reported that higher violations were related to the bus drivers’ accident involvement. Bener et al. [39] showed that higher scores of errors, aggression-speeding, and lapses were positively related to accident involvement. 

### 2.4. Contribution of the Current Study 

The literature suggests that (1) most of the previous studies have been conducted on samples obtained from the general driving population, including car, bus, and truck drivers; (2) a large body of studies has examined the DBQ in Western and European countries and areas with good safety performances, such as Denmark [40], the United Kingdom [36], France [41], New Zealand [23], Australia [26,32], and North America [25]; and (3) most of the previous studies have limited themselves to an examination of the correlation between a few demographic variables (e.g., age and gender) and driving characteristics (e.g., annual mileage), and DBQ factors and accident risks. Despite extensive review, we did not find any study that focused on the DBQ factors of taxi drivers in a Middle Eastern context. Machin and De Souza [42] showed that 29.7% of the accidents were caused by unsafe driving behaviours of taxi drivers in Australia. Also, they found that the major variables contributing to the unsafe driving behaviours were aberrant driving behaviours and personal factors. Hence, this study contributes to the literature by examining a wide range of socioeconomic variables in predicting high scores of the DBQ factors and accident risks among Iranian taxi drivers.

### 2.5. Aims and Hypotheses

The aim of this study was to examine the association between different background variables and the DBQ factors of Iranian taxi drivers, and their accident involvement and receiving traffic tickets. It was hypothesized that the drivers with different taxi driving experiences, demographics, and socioeconomic features might display different aberrant driving behaviour (e.g., lapses and ordinary violations). For example, well-educated and older drivers often commit fewer violations. We were also interested in investigating the role of a several variables such as years of taxi driving experience, household size, and economic status of drivers (e.g., income status and car ownership), as well as the DBQ factors in the accident involvement and traffic tickets. 

## 3. Materials and Methods

### 3.1. Procedure and Respondents

A sample of Iranian taxi drivers was recruited during October and November 2016. A cross-sectional design using a self-completion questionnaire survey was employed for data collection in this study. Based on local data resources such as the number of taxi drivers and taxi stations in the urban network, 20 taxi stations were selected for data collection in two Iranian cities. The study areas were in Bojnurd and Neyshabur in the northeast of Iran. Bojnurd and Neyshabur have a population of 324,083 and 451,780, respectively [43]. Public transport (PT) in the cities includes urban buses and taxis (shared taxi). The taxis have specific stations (origins and destinations) across the cities. However, they do not have dedicated paths and hence drive on all main streets and roads similar to other private vehicles in the cities. The taxi stations are usually fixed-point waiting locations for picking up and dropping off passengers. Taxis usually have a capacity of four passengers and as soon as a taxi picks up its four passengers, the driver heads for the destination. The selected cities have some interesting features, which motivated us to select them for in this study. For example, their public transport system and the general urban traffic patterns are representative of most Iranian cities and other less developed regions in the Middle East.

A convenience sampling method was employed to collect data in the taxi stations. Of the 405 distributed questionnaires among taxi drivers in the selected stations, 24 had not answered the questions relevant to risk involvement and DBQ. Hence, these cases were removed from the analysis, leaving 381 valid observations for further analysis. It is important to note that almost all of the Iranian taxi drivers are males. Therefore, the present sample only included male taxi drivers. 

Participation in the study was on an anonymous and voluntary basis. The taxi drivers were ensured that the survey and responses would have no influence on their driving assessment by the traffic police or taxi industry management. They were also assured that no information would be delivered to their employing firms.

### 3.2. Measures

#### 3.2.1. Demographic, Socioeconomic, and Driving Characteristics

Information regarding the drivers’ demographic and socioeconomic variables and driving characteristics were gathered in the first part of the questionnaire. The age, educational background (above high school = 1, high school or lower = 0), marital status (single = 1, other = 0), household size, car ownership status, and income level of the participants were recorded. The driving characteristics including the annual mileage (km), years of driving’ experience, and hours of driving in a week, and the number of respondent’s daily taxi trips were also recorded. 

#### 3.2.2. Driver Behaviour Questionnaire Measurement 

The validated 27-item DBQ [17,19] was used to measure aberrant driving behaviour. The original version of DBQ in Persian, which has been used in this study, was already tested and validated on a group of professional lorry drivers in Iran [20]. The content validity of the original questionnaire was checked by a panel of experts. In addition, before data collection, a pilot survey was conducted among 30 taxi drivers in the study area, to examine whether the survey instruments and procedures yielded the desirable outcomes. The pilot study led to minor corrections in the questionnaire. For instance, the wording of some background variables was revised and one DBQ-item was changed. The original English items were translated into Persian by two native Persian co-researchers; these were then translated back into English by another English expert. A six-point Likert scale (0 = never, 1 = hardly ever, 2 = occasionally, 3 = quite often, 4 = frequently, and 5 = nearly all the time) was used for measuring all of the items. The taxi drivers were questioned to report how often they had engaged in each of the 27 behaviours in the past year. The instrument included eight items relevant to lapses (L) like, “Realise that you have no clear memory of the road you have been travelling on”. Aggressive violations (AV) contained six items, such as, “Become angry at another driver and chase them with the intention of showing them how angry you are”. Ordinary violations (V) included five items, such as, “Disregard the speed limit on a residential road”. Errors (E) also contained eight items, such as, “When turning left, nearly hit a bicycle rider who has come up on your left”. 

#### 3.2.3. Risk Involvement

The information regarding the taxi drivers’ accident involvement and the number of traffic tickets was queried. The survey assessed how many accidents the taxi drivers had been involved in during the last year. The definition of accidents also covered injury to the participant (and any other person, such as pedestrians) and damage to property or vehicles [23]. The number of received traffic tickets of the taxi drivers was also recorded for the past year.

### 3.3. Statistical Analysis

To reveal the profiles of the background variables (demographic, socioeconomic, and driving characteristics) and the overall scores of the DBQ items, descriptive statistics were applied. To test the DBQ’s dimensional structure, the instrument principal component analyses (PCA) with a varimax rotation and iteration were applied. To determine the number of extracted components, the Scree-plot and Kaiser criterion (an eigenvalue above 1.00 was considered to be a significant value) were used. A factor loading above 0.40 was used as a criterion for items to be retained in the DBQ components. To test the internal consistency and reliability of the scales, Cronbach’s α (alpha) was calculated. Furthermore, the average corrected inter-item total correlations (Aiic) were calculated as indicators of reliability, because Cronbach’s α tends to be biased when the scales contain few or many items [44]. The cut-off value of 0.30 was considered for the Aiic. In addition to the PCA, a confirmatory factor analysis (CFA) was utilized to confirm the factors identified in the current study and in the literature. The factor structure of the 27-item DBQ was confirmed using CFA with Amos 23. The root mean square error of approximation (RMSEA), the comparative fit index (CFI), and the Tucker–Lewis index (TLI) were applied as fit indices to determine the fitness of the data with the specified model [45]. RMSEA values below 0.06 and CFI and TLI values between 0.90 and 0.95 reflected an adequate fit [46]. The chi-square (χ^2^) with corresponding significance level was also reported.

To predict the scores of the identified DBQ-factors across the background variables (demographic, socioeconomic variables, and driving characteristics), four linear regression models were employed. The multiple linear regression model with the *k* predictor variables (*x*_1_, *x*_2_, *x*_3_, …, *x_κ_*) and a continuous (or interval scale) dependent variable, *y*, can be written as Equation (1), as follows:

(1) $$y=\beta_{0}+\beta_{1}x_{1}+\beta_{2}x_{2}+\ldots \beta_{k}x_{k}+\varepsilon$$

where ε is the residual terms of the model and β*_i_* is the regression coefficient. The ordinary least squares method was used for estimation. Furthermore, two binary logistic regression models were applied to predict the accident involvement and received traffic tickets. The binary logistic regression model is a type of predictive modelling that can be applied when the dependent variable is binary; that is, when there are only two possible outcomes (e.g., accident involvement = 1, otherwise = 0). The general form of the logistic regression model is Equation (2), as follows:

(2) $$\log(\frac{p_{1}}{1-p_{1}})=\beta_{0}+\beta_{1}x_{1}+\beta_{2}x_{2}+\ldots \beta_{k}x_{k}$$

where, *p*_1_ is the probability that *x*_1_, *x*_2_, *x*_3_, …, *x_κ_* are predictors, and β*_i_* is the regression coefficient. To estimate this model, the maximum likelihood method was employed.

Furthermore, the dichotomous variable (yes/no) about accident involvement and ticket involvement was defined as the dependent variable in the binary logistic models. The dependent variable of the self-report accident involvement was set to 1 if the drivers had been involved in at least one accident over the past year, and zero otherwise (no accident). Furthermore, the dependent variable for the self-report traffic tickets was set to 1 if they had received at least one traffic ticket during the past year, and zero otherwise (no ticket).

In addition to the variables found statistically significant in a 95% confidence interval, all of the non-significant tested variables have been adjusted for and been reported in the models. To calculate the marginal effect of each explanatory variable on the outcome variable, the odds ratio (OR) was reported. The OR for an explanatory variable indicates the relative amount by which the odds of a dependent variable increase (OR >1) or decrease (OR <1) when the value of the explanatory variable is increased by 1.0 unit. These statistical analyses were carried out in SPSS 22.0.

## 4. Results

### 4.1. Characteristics of the Driver Behaviour Questionnaire Items

Table 1 shows the descriptive statistics of the background variables. On average, the taxi drivers were 36.26 years old (standard deviation (SD) = 11.57). The mean annual driving mileage was 79,143.31 km (SD = 72875.12) (see Table 1). The drivers had an average of 81.32 (SD = 62.67) hours of driving in a week, and 13.42 (SD = 14.21) years of experience driving a taxi. On average, 38.03% of the respondents reported that they had been involved in at least one accident during the last year.

Table 2 shows the means and standard deviations of all of the DBQ items reported by the taxi drivers. Among the 27 DBQ items, on the aggressive violation item, “Get angry at a certain type of driver and express your anger any way you can” (mean (M) = 2.24, SD = 1.97) and an ordinary violation item, “Drive so close to the car in front that it would be difficult to stop in an emergency” (M = 2.11, SD = 1.78), were the highest reported aberrant driving behaviours (Table 2). The item, “Misread signs and exit roundabout on the wrong road” (M = 1.43, SD = 1.32) was the highest reported lapse among taxi drivers. Furthermore, the item, “Queuing to turn left onto a main road, you pay such close attention to the traffic on the main road that you nearly hit the car in front” (M = 2.01, SD = 1.69) was the most prevalent reported error.

### 4.2. Dimensionality and Reliability Indices of the Driver Behaviour Questionnaire Items 

The PCA with iteration and varimax rotation showed that the DBQ segmented into four dimensions in the sample of the Iranian taxi drivers. Table 3 shows the results of the PCA solution. This solution explained 55.12% of the variance. The first dimension, the errors, included eight items (Cronbach α = 0.76, average corrected inter-item correlation = 0.61, explained variance = 22.33%). Ordinary violations, the second dimension, contained six items (Cronbach α = 0.83, average corrected inter-item correlation = 0.69, explained variance = 13.47%). The third dimension, lapses, included four items (Cronbach α = 0.71, average corrected inter-item correlation = 0.50, explained variance = 10.77 %). The fourth dimension, the aggressive violations, included three items (Cronbach α = 0.74, average corrected inter-item correlation = 0.51, explained variance = 8.55%). The results of a CFA for the DBQ displayed good fitness (χ^2^ = 750.14, degree of freedom = 203, *p* < 0.001, RMSEA = 0.052, CFI = 0.92, TLI = 0.92) with a four-factor structure, which was similar to the PCA results. As for the distribution, all of the variables used in the CFA were shown to have normal distribution. Furthermore, the Shapiro–Wilk (S–W) test for all of the variables did not indicate any significant deviation from normality.

### 4.3. Predictors of the Driver Behaviour Questionnaire Factor Scores 

Table 4 shows that four multiple linear regression models significantly predicted four factors of the DBQ as four dependent variables (DV) (e.g., the model summary for the model with DV ‘error’ *F-value* = 2.20, *p <* 0.01, *R*^2^ = 0.11, *Adjusted R*^2^ = 0.08). Older taxi drivers reported more errors (Beta (*B*) = 0.24, *p* < 0.001), and lower ordinary (*B* = −0.83, *p* < 0.05) and aggressive violations (*B* = −0.48, *p* < 0.001). A higher educational background had a negative effect on aggressive violations (*B* = −0.82, *p* < 0.001). Drivers who were single were more likely to commit ordinary (*B* = 0.15, *p* < 0.01) and aggressive violations (*B* = 1.21, *p* < 0.001). A higher level of car ownership (*B* = −0.25, *p* < 0.05) and income (*B* = −0.41, *p* < 0.05) were positively associated with more aggressive violations. A higher annual driving mileage was related to more ordinary (*B* = 0.51, *p* < 0.001) and more aggressive violations (*B* = 0.28, *p* < 0.05). Furthermore, more hours of driving were positively associated with greater violations, while more years of driving experience were correlated with fewer violations. The taxi drivers who had more daily trips tended to report more violations.

### 4.4. Predictors of the Risk Involvement

Table 5 shows that two binary logistic models significantly predicted accident involvement (Model χ^2^ = 203.91, *p* < 0.001) and traffic tickets (Model χ^2^ = 198.12, *p* < 0.001). The old age of taxi drivers (OR = 0.78, *p* < 0.001) and high-income level of households (OR =0.86, *p* < 0.05) were negatively related to a traffic accident. The results showed that driver’s marital status (OR = 1.61, *p* < 0.001), higher annual driving mileage (OR = 2.12, *p* < 0.001), higher hours of driving (OR = 1.75, *p* < 0.001), and higher number of daily taxi trips (OR = 1.83, *p* < 0.001) were positively associated with the accident involvement. Regarding the relationship between the DBQ-factor and accident involvement, more ordinary violations (OR = 1.63, *p* < 0.001) and aggressive violations (OR = 1.92, *p <* 0.001) were positively related to accident involvement. 

The results also showed that being a single driver (OR = 1.85, *p* < 0.05), having a higher annual mileage (OR = 2.75, *p* < 0.001), higher hours of driving in a week (OR = 2.10, *p* < 0.001), a higher number of daily trips (OR = 1.48, *p* < 0.001), more errors (OR = 1.34, *p <* 0.001), and more ordinary violations (OR = 1.28, *p <* 0.05) were positively associated with receiving traffic tickets. Also, a higher level of car ownership (OR = 0.83, *p* < 0.05) and higher income level (OR = 0.92, *p* < 0.05) were negatively related to traffic tickets involvement.

## 5. Discussion 

The purpose of the current study was to examine the predictors of risk involvement and aberrant driving behaviour among Iranian taxi drivers. The study attempted to identify the barriers of public health in the professional or occupational context of taxi drivers.

Firstly, in accordance with several previous studies that have been conducted among the general driving population of car, bus, and truck drivers in other countries, the current study identified and confirmed a four-factor solution of the DBQ for taxi drivers [19,24,26]. Secondly, the study explored several predictors of drivers’ aberrant driving behaviours. Thirdly, the study examined whether the demographic, socioeconomic, driving characteristics, and the DBQ factors (error, lapse, ordinary violation, and aggressive violation) could predict two outcomes of risk involvement (accident and traffic tickets).

The majority of studies conducted on the general driving population in countries with a Western cultural orientation showed that some of the DBQ factors, such as ordinary violation and demographic characteristics (e.g., age), were predictors of accident involvement [15]. In contrast to several previous studies, and in accordance with the hypotheses of the study, the findings of the current study showed that several socioeconomic variables and aberrant driving factors were significant predictors of risk involvement among Iranian taxi drivers. The findings indicated that, in addition to ordinary violations, the reported errors and aggressive violations of taxi drivers could be seen as significant and positive predictors of risk involvement in Iran. These findings support our hypotheses that drivers with different taxi driving experiences, demographics, and socioeconomic features as well as different DBQ factors might influence risk involvement among taxi drivers.

Somewhat expectedly, the mean scores of the DBQ-items, including items relevant to ordinary violation such as, “drive so close to the car in front that it would be difficult to stop in an emergency”, and aggressive violations such as, “get angry at a driver and express your anger any way you can”, were higher among Iranian taxi drivers compared with the general driving population [24,26], bus drivers [37], and truck drivers [23] in Western countries. A plausible explanation may be the nature of the study population (urban taxi drivers versus general public). In particular, a higher exposure of Iranian taxi drivers and also more hours of driving in urban road traffic influenced aberrant driving behaviours. A potential explanation for this relationship may be the negative influence of factors related to driver’s work-related physical and mental characteristics, such as fatigue, sleepiness, anger, and stress [9,47]. Additionally, heavy traffic, narrow streets, the overall chaotic driving environment in the study area, delays and traffic congestion might increase such mental and physical features. Nordfjærn et al. [48] also speculated that professional drivers might perceive significantly more control and be involved in more accidents compared with non-professional drivers. This suggests that professional drivers like taxi drivers may constitute a risk group in the road traffic.

Regarding the relationship between the aberrant driving behaviour and different background variables, the four DBQ factors were significantly predicted by several explanatory variables, such as the age and income status of drivers. In contrast to several previous studies [15], and in line with the results of Tabibi et al. [49] and the hypotheses of this study, the findings showed that older taxi drivers were more likely to commit driving errors. Interestingly, high-income drivers were less likely to commit aggressive violations. To decrease violations, the finding highlights that policymakers should consider the income status of taxi drivers, such as the development of taxi trade unions and developing salary regulations in Iran. Additionally, single taxi drivers were more likely to report ordinary and aggressive violations. These drivers might generally be more likely to engage in risk-taking behaviours and excitement-seeking behaviours [50].

In line with previous studies [23,24], the probability of involvement in a traffic accident tended to increase with the more reported ordinary violations. In addition to ordinary violations, the study found that more aggressive violations of taxi drivers were strong and positive predictors of accident involvement. This study also investigated the predictors of other measures of risk involvement (i.e., receiving traffic ticket). More errors and ordinary violations were positively related to greater traffic ticket involvement. 

Among the background variables, the young age of taxi drivers was associated with involvement in accidents, which is congruent with the literature [23]. The authors found the positive influence of young age on the higher accident involvement of truck drivers in New Zealand. Single drivers are more likely to be involved in an accident and receive traffic tickets. Previous findings have also shown that single drivers are more prone to risk-taking behaviours (e.g., [51]). The findings also showed that a higher annual mileage, higher hours of driving, and a higher number of daily trips were related to higher accidents and tickets. Lourens et al. [31] also showed that accidents were positively correlated with annual mileage (exposure) in the general driving population in the Netherlands. Policymakers could aim to reduce accident involvement of their taxi drivers by controlling the road traffic exposure among the drivers. 

To summarise, among the different demographic, socioeconomic, and driving factors, being a young and single driver alone, with factors such as a high income, high annual driving mileage and hours of driving, higher number of daily taxi trips, and reported ordinary and aggressive traffic violations were positively related to a higher accident involvement among taxi drivers. However, educational background, household size, car ownership status, driving experiences, errors, and lapses were not associated with accident involvement. Furthermore, the non-ownership of the cars, low income, high annual driving mileage and hours of driving, high number of daily taxi trips, more errors, and ordinary traffic violations were positively associated with receiving more traffic tickets. Meanwhile, the age of drivers, educational background, household size, driving experiences, lapses, and aggressive violations did not significantly affect the relationship between the explanatory variables and receiving traffic tickets.

### Limitations

The present study has limitations such as the self-reported nature of measurements, the cross-sectional design, and convenience sampling. The questionnaire was based on self-reports including demographic variables, socioeconomic characteristics, risk involvement, and psychological instruments such as aberrant driving behaviour (the 27-item DBQ). This may impose limitations regarding potential socially desirable responses, causal explanations between the variables, and issues regarding representativity. However, in the present study, we examined a rather large sample (10% of the study population) of taxi drivers scattered in all regions of Bojnurd and Neyshabur (two cities in Iran). This may increase the likelihood of a representative sample. A great deal of underreporting and forgetting about driving accidents has been pointed out in a study [24]. This issue also reduces the strength of any potential relationship and the variability in accident involvement.

## 6. Conclusions

Much of the previous research on aberrant driving behaviour has focussed on examining the factor-solution of the DBQ among the general driving population in Western settings. Little evidence exists to investigate a wide-range of demographic variables, socioeconomic characteristics, and driving factors on the DBQ factors, as well as on the accident involvement among taxi drivers as public transport drivers in the Middle East context. This research augmented the literature by exploring the predictors of the DBQ factors and the risk involvement among Iranian taxi drivers. The study explored a four-factor solution of a 27-item DBQ including errors, ordinary violations, lapses, and aggressive violations. The study further indicated that several variables such as the drivers’ age, marital status, annual mileage, number of daily trips, and ordinary and aggressive violations could influence accident involvement.

The findings of the current study could be utilized for implementing and planning safe driving behaviour interventions among taxi drivers. For example, interventions aimed to enhance the traffic safety of taxis, as public transport, could tackle these risk by careful management of the work schedules, including the number of daily trips, hours of driving (see [52]), and improving the economic conditions of taxi drivers [5]. This encourages more country-specific studies in the future. This may also require an attitude change in the taxi industry management, where safety is equally important or prioritized over economic profits and efficiency. Furthermore, establishing improved training and qualification mechanisms for taxi drivers could be implemented by traffic safety experts to reduce the ordinary and aggressive violations among the specific groups of taxi drivers, such as young and single drivers.

## Figures and Tables

**Table 1:** Descriptive statistics of the background variables and risk involvement (*n* = 381). SD—standard deviation.

Variable	Description	Mean	SD
Age of driver	Continuous variable	36.26	11.57
Educational background of driver	High (higher than high school) = 1, low = 0	0.37	0.47
Driver’s marital status	Single = 1, otherwise = 0	0.14	0.34
Driver’s household size	Number	2.90	1.82
Number of owned private car in household	Number	0.94	0.35
Driver’s income status	Higher than two million Tomans * = 1, otherwise = 0	0.14	0.35
Annual driving mileage	Continuous variable (kilometres in the last year)	79,143	72,875
Hours of driving in a week	Continuous variable (hours in a week)	81.32	62.67
Years of driving experience of a taxi	Continuous variable (unit: year)	13.42	14.21
Number of respondent’s taxi trips in each day	Number	47.34	45.57
Accident involvement in the last year (self-reported)	At least one accident = 1, no accident = 0	0.38	0.48
Traffic tickets involvement in the last year (self-reported)	At least one ticket = 1, no ticket = 0	0.43	0.49

* 1 Euro = 3496 Tomans (August 2017).

**Table 2:** Means and standard deviations of all Driver Behaviour Questionnaire (DBQ) items reported by the taxi drivers (*n* = 381).

Item	Mean	SD
**Errors (E)**		
Queuing to turn left onto a main road, you pay such close attention to the traffic on the main road that you nearly hit the car in front	2.01	1.69
Fail to notice that pedestrians are crossing when turning into a side street from a main road	1.97	1.43
Brake too quickly on a slippery road	1.85	1.27
Fail to check your rear-view mirror before pulling out, changing lanes, etc.	1.52	1.33
When turning left, nearly hit a bicycle rider who has come up on your left	1.21	1.06
Attempt to overtake someone that you had not noticed was signaling a right turn	0.97	1.10
Miss seeing a “give way” sign and just avoid colliding with traffic having the right of way	0.83	0.97
Underestimate the speed of an oncoming vehicle when overtaking	0.67	0.88
**Lapses (L)**		
Misread signs and exit roundabout on the wrong road	1.43	1.32
Switch on one thing, such as the headlights, when you meant to switch on something else, such as the wipers	1.24	1.32
Attempt to drive away from traffic lights in the wrong gear	0.91	1.19
Forget where left taxi/car in a taxi/car park	0.78	0.96
Having set out to drive to one place, you suddenly realize you are on the road to somewhere else	0.75	1.03
Get in the wrong lane approaching a roundabout or junction	0.69	0.90
Realize that you have no clear memory of the road you have been travelling on	0.64	0.87
Hit something when reversing that you had not previously seen	0.58	0.94
**Ordinary violations (OV)**		
Drive so close to the car in front that it would be difficult to stop in an emergency	2.11	1.78
Enter an intersection knowing that the traffic lights have already changed against you	1.91	1.66
Overtake a slow driver on the inside	1.45	1.55
Disregard the speed limit on a residential road	1.31	1.22
Disregard the speed limit on a freeway or rural highway	0.96	1.05
**Aggressive violations (AV)**		
Get angry at a certain type of driver and express your anger any way you can	2.24	1.97
Use your horn to indicate your annoyance to another road user	2.03	1.84
Become angry at another driver and chase them with the intention of showing them how angry you are	1.93	1.75
Stay in a lane that you know will be closed ahead until the last minute before forcing your way into the other lane	1.79	1.52
Race away from traffic lights with the intention of beating the driver next to you	1.73	1.44
Pull out of an intersection so far you force your way into the traffic	0.93	1.01

**Table 3:** Principal component analysis (PCA) for the DBQ items.

Dimensions	Loadings			
	Errors	Ordinary Violations	Lapses	Aggressive Violations
**1—Errors (Cronbach α = 0.765, Aiic = 0.61, Ev = 22.33%, Dimension’s mean (SD) = 1.38 (0.50))**				
Fail to notice that pedestrians are crossing when turning into a side street from a main road (E)	0.82			
Attempt to overtake someone that you had not noticed was signalling a right turn (E)	0.76			
Queuing to turn left onto a main road, you pay such close attention to the traffic on the main road that you nearly hit the car in front (E)	0.73			
Miss seeing a “give way” sign and just avoid colliding with traffic having the right of way (E)	0.71			
Fail to check your rear-view mirror before pulling out, changing lanes, etc. (E)	0.70			
When turning left, nearly hit a bicycle rider who has come up on your left (E)	0.64			
Brake too quickly on a slippery road (E)	0.59			
Get in the wrong lane approaching a roundabout or junction (L)	0.41			
**2—Ordinary violations (Cronbach α = 0.832, Aiic = 0.69, Ev = 13.47%, Dimension’s mean (SD) = 1.40 (0.50))**				
Drive so close to the car in front that it would be difficult to stop in an emergency (OV)		0.74		
Enter an intersection knowing that the traffic lights have already changed against you (OV)		0.70		
Disregard the speed limit on a freeway or rural highway (OV)		0.65		
Disregard the speed limit on a residential road (OV)		0.61		
Overtake a slow driver on the inside (OV)		0.55		
Underestimate the speed of an oncoming vehicle when overtaking (E)		0.46		
**3—Lapses (Cronbach α = 0.710, Aiic = 0.50, Ev = 10.77%, Dimension’s mean (SD) = 0.92 (0.34))**				
Misread signs and exit roundabout on the wrong road (L)			0.68	
Hit something when reversing that you had not previously seen (L)			0.62	
Realise that you have no clear memory of the road you have been travelling on (L)			0.57	
Having set out to drive to one place, you suddenly realise you are on the road to somewhere else (L)			0.51	
**4—Aggressive violations (Cronbach α = 0.742, Aiic = 0.51, Ev = 8.55%, Dimension’s mean (SD) = 2.06 (0.13))**				
Use your horn to indicate your annoyance to another road user (AV)				0.74
Get angry at a certain type of driver and express your anger any way you can (AV)				0.71
Become angry at another driver and chase them with the intention of showing them how angry you are (AV)				0.63

Notes: Factor loadings <0.40 not reported. E—error; L—lapse; OV—ordinary violation; AV—aggressive violation; Aiic—average corrected inter-item correlation; Ev—explained variance.

**Table 4:** Predictors of the four DBQ factors.

Variable	Error		Ordinary Violation		Lapses		Aggressive Violation	
	B	*t*-Test	B	*t*-Test	B	*t*-Test	B	*t*-Test
Constant	2.05	117	3.05 ***	3.89	−2.21	−1.04	2.76	1.43
Age of driver	0.24 ***	3.81	−0.83 **	−2.45	−0.09	−1.27	−0.48 ***	−3.46
Educational background of driver	0.13	0.92	−0.22	−1.50	0.17	1.34	−0.82 ***	−3.02
Driver’s marital status	−0.33	−1.40	0.15 *	2.22	−0.04	−0.42	1.21 ***	4.21
Driver’s household size	0.16	1.02	−0.08	−1.25	0.12	1.31	−0.11	−1.84
Number of owned private car in household	0.07	1.32	−0.12	−1.76	−0.05	−0.42	−0.25 *	−2.21
Driver’s income status	0.18	1.09	−0.30	−1.60	0.02	0.19	−0.41 *	−2.23
Annual driving mileage	−0.37	−1.51	0.51 ***	3.12	0.14	1.25	0.28 *	2.19
Hours of driving in a week	−0.08	−0.97	0.71 ***	3.80	−0.57***	−3.52	0.89 ***	4.12
Years of driving experience of a taxi	−0.10	−1.30	−0.45 *	−2.21	−0.23	−1.34	−0.13 *	−2.15
Number of respondent’s taxi trips in each day	0.12	1.41	0.94 ***	3.91	−0.12	−1.11	1.51 ***	4.83
Model summary	*F* = 2.20 **, *R*^2^ = 0.11, *Adjusted R*^2^ = 0.08		*F* = 3.91 ***, *R*^2^ = 0.27, *Adjusted R*^2^ = 0.18		*F* = 2.18 **, *R*^2^ = 0.10, *Adjusted R*^2^ = 0.07		*F* = 2.20 **, *R*^2^ = 0.11, *Adjusted R*^2^ = 0.08	

*: *p* < 0.05, **: *p* < 0.01, ***: *p* < 0.001. B—regression coefficient of variables in the model.

**Table 5:** Predictors of the accident involvement and receiving traffic tickets.

Variable	Accident Involvement			Tickets Involvement		
	B	OR	Wald	B	OR	Wald
Constant	1.18	-	1.23	3.19 ***	-	10.45
Age of driver	−0.25 ***	0.78	6.12	−0.11	0.89	1.48
Educational background of driver	−0.12	0.92	1.57	−0.08	0.93	1.39
Driver’s marital status	1.27***	1.61	9.42	1.34 *	1.85	4.38
Driver’s household size	0.11	1.08	1.35	0.09	1.02	1.12
Number of owned private car in household	0.24	0.91	1.55	−0.34 *	0.83	4.84
Driver’s income status	−0.12*	0.86	4.31	−0.08 *	0.92	4.27
Annual driving mileage	0.80 ***	2.12	12.44	0.93 ***	2.75	10.91
Hours of driving in a week	0.54 ***	1.75	11.21	0.63 ***	2.10	13.41
Years of driving experience of a taxi	0.13	1.24	1.61	0.24	1.12	1.39
Number of respondent’s taxi trips in each day	0.64 ***	1.83	10.34	0.53 ***	1.48	13.11
Error (factor1)	0.34	1.21	1.67	0.25 ***	1.34	14.21
Ordinary violation (factor2)	0.71 ***	1.63	12.01	0.18 *	1.28	4.41
Lapse (factor3)	0.07	1.19	1.20	0.11	1.04	1.13
Aggressive violation (factor4)	0.81 ***	1.92	11.67	0.29	1.12	1.71
Model summary	Chi-square = 203.91 (degree of freedom (df) = 15), enter method, sig = 0.000. *R*^2^ = 0.28 (Cox and Snell), 0.43 (Nagelkerke). 82.3% correctly predicted, HL: Chi-square (df = 8) = 4.80, sig = 0.779.			Chi-square= 198.12 (df = 15), enter method, sig = 0.000. *R*^2^ = 0.21 (Cox and Snell), 0.39 (Nagelkerke). 71.2% correctly predicted, HL: Chi-square (df = 8) = 3.57, sig = 0.628.		

*: *p* < 0.05, **: *p* < 0.01, ***: *p* < 0.001. B—regression coefficient of variables in the model. OR—odds ratio; the OR for an explanatory variable tells us the relative amount by which the odds of a dependent variable increase (OR >1) or decrease (OR <1) when the value of the explanatory variable is increased by 1.0 unit. Wald—the Wald test is the test of significance for individual regression coefficients in logistic regression. HL: Hosmer–Lemeshow test.
